# Comparing Health Promotion Programs in Public Dental Service of Vantaa, Finland: A Clinical Trial in 6–36-Month-Old Children

**DOI:** 10.1155/2013/757938

**Published:** 2013-11-18

**Authors:** Irma Arpalahti, Mimmi Tolvanen, Kaisu Pienihakkinen

**Affiliations:** ^1^Health and Social Welfare Affairs, Pakkalankuja 5, 01510 Vantaa, Finland; ^2^Department of Community Dentistry, Institute of Dentistry, University of Turku, Lemminkaisenkatu 2, 20014 Turku, Finland

## Abstract

*Objective*. The study assessed whether the new family-based programs in health promotion or the training of dental professionals had an impact on the colonization of mutans streptococci (MS) in young children. *Material and Methods*. The participants were children born in 2008 and inhabitants of Vantaa aged 24–36 months. The families with first-born children were invited to a questionnaire study. Vantaa was categorized into three matching areas, which were randomly assigned to different programs. New counseling methods were trained. The routine program used earlier served as the control group. The children born in 2006 served as a historic control. The outcome measure was the presence of MS. Statistical method was logistic regression. *Results*. Colonization of MS was found only in few children born in 2006 or 2008; 15% and 11%, respectively. Within the 2008 birth cohort, the addition of parental counseling did not improve the routine program. Instead, the father's advanced level of education (*P* = 0.044) and the child's reported the use of xylitol at least three times a day (*P* = 0.014) associated with negative MS scores. *Conclusions*. The routine program and training of the professionals seem to reduce the proportion of children with MS more than adding parental self-care to oral health programs.

## 1. Introduction

A three-year health promotion project was started in the Vantaa PDS in 2006. The main reason for the project was that the oral health of children and adolescents had been deteriorating. For prevention of early childhood caries, the oral health promotion was started earlier than before by giving health advice to expecting parents. In addition, the training of modern methods in counseling was targeted at the professionals involved in children's oral health care. The main aim of the project was to delay the transmission of mutans streptococci (MS) from parent to child. The present study was initially started in order to evaluate the achievements of the project. 

Oral health promotion in early childhood includes preventive work aimed at expecting families, babies, and young children. According to results from recent studies, this also seems to be effective and justified [1–3]. The Scottish Intercollegiate Guidelines Network recommends that programs of oral health promotion should be available to parents during pregnancy to reduce early childhood caries, and that the counseling for parents and their preschool children should start before the age of three [4]. The Finnish current care guideline for caries control emphasizes early childhood health promotion and acquisition of favorable oral health habits [5]. Oral health counseling in early childhood increases the knowledge of parents and decreases the incidence of early childhood caries [6]; the counseling is particularly important in areas with a high prevalence of caries. 

Dental plaque is a biofilm that is naturally found on the teeth. MS are transmitted mainly from caregivers to children in early childhood and they colonize tooth surfaces, but they can also be present in the edentulous mouth of a young child on soft tissues and tongue [7]. High proportions of MS may be considered biomarkers of rapid caries progression [8], and in young children, the colonization of MS in saliva or plaque is connected to increased risk of caries [9]. Colonization of MS in early childhood has been connected to the lower socioeconomic status of families [10]. In the early risk-based approach, preventive counseling and individual caries-controlling measures can be carried out in time and have been found to be health-effective and cost-effective [1].

It is important how the health information is delivered. Patients benefit more from interactive methods in counseling instead of the traditional professional-oriented method. The transtheoretical model (TTM) [11] helps to determine the individual level of decisional balance and to focus on individual conversation and goal setting in counseling. In the TTM, the intention to take action in changing health behavior increases from the stage of precontemplation to contemplation and preparation. In the action stage, patients have already accepted the new health habits but they are still at risk of relapsing. Understanding the fluctuations between stages of change may also decrease the frustration of the counselor if the patient is not ready to change his/her behavior or has a relapse. In the method of motivational interviewing (MI) [12, 13], the feeling/meaning reflections, change talk, and goal setting are the core elements. By means of empathy, congruence, and positive regard, which create a feeling of acceptance, patients are free to make changes in their behaviors [14]. Patients seem to benefit from combining the TTM and MI [15]; the health care professional can give individual counseling tailored to each patient's stage of change. 

We hypothesized that the training of dental professionals could improve the quality of the counseling, raise the level of commitment, and give them a new kind of perspective on oral health counseling in early childhood. Health promotion programs with clinical advice aimed at the parents themselves might commit the families to maintain their children's good oral health.

The present study assessed whether the new family-based programs in oral health promotion or the training of dental professionals had an impact on the colonization of mutans streptococci in young children, when the other protective factors were controlled for.

## 2. Methods

### 2.1. Study Settings

The study took place in the public dental service (PDS) of Vantaa, Finland, in 2008–2011. The routine oral health program in use earlier was compared with two new programs, carried out at children's routine oral health visits from 6 to 36 months of age. There were 28–35 dental hygienists or in-service trained dental nurses (professionals) involved in children's counseling during the study, and they were trained to use the new oral health programs and novel methods in counseling. The professionals were not blinded to the group of children but the interpretation of the children's MS test results was blinded. The ethical committee of the hospital district of Helsinki and Uusimaa gave ethical approval for this study. The identifier in ClinicalTrials.gov is NCT01854502.

### 2.2. Randomization

The five operational areas of the Vantaa PDS were categorized into three study areas that were socioeconomically as comparable as possible. The two western areas were previously known to be better off than the eastern areas; therefore, two of the areas were selected to include both western and eastern areas of Vantaa, and the central area formed the third area. These three matched areas were randomly allocated to three study groups, F (oral hygiene and fluoride), X (proper diet and xylitol), and C (control), by drawing lots. 

### 2.3. Subjects and Recruitment

**Figure 1:**
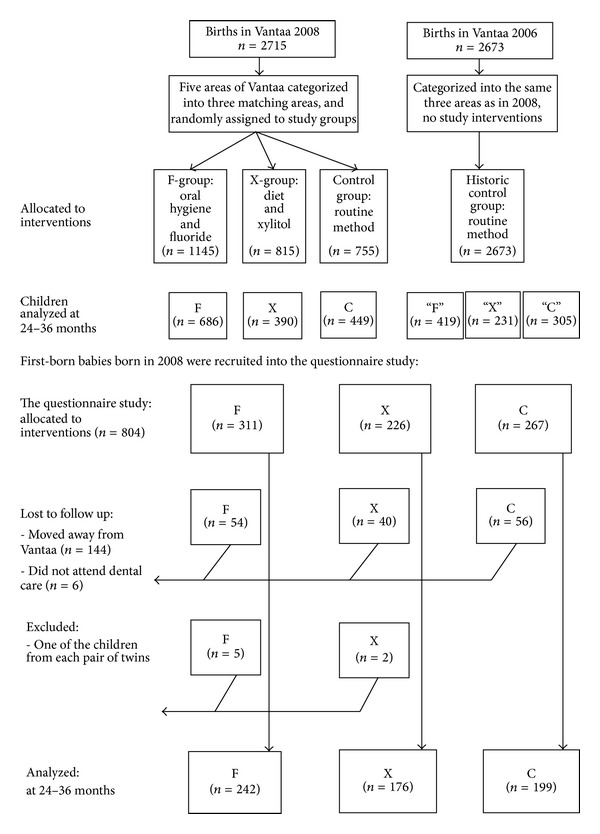
The flow chart: those allocated to interventions, those recruited into the questionnaire study, and those analyzed.

The subjects in this study were the children born in 2008 (*n* = 2715) and inhabitants of Vantaa aged 24–36 months. Additionally, the first-born babies of the 2008 birth cohort were invited to join the questionnaire study when the baby was 2–4 weeks old. The invitation letter included an informed consent form for the parents on behalf of the minor, and 804 (82%) of the first-born babies joined the study (Figure 1). The first-born babies were selected in order to control the confounding effects of the number of siblings and the order of the child in the family. There were seven pairs of twins and the child with the higher social security number from each pair was withdrawn from the questionnaire study. If the child moved away from Vantaa (*n* = 144) or did not attend public dental care (*n* = 6), he or she was lost to follow up. In connection with the invitation to the two-year examination, the parents were sent a questionnaire concerning their own background information and the health habits of their child. Additionally, the children born in 2006 (*n* = 2673) and inhabitants of Vantaa aged 24–36 months served as a historic control group. They were categorized retrospectively for data collection according to the same areas as the children born in 2008, but were not given any special interventions. 

The calculations of sample size were based on the hypothesis that 30% of two-year-olds have MS in dental plaque [16]. The 2008 birth cohort consisted of 2715 children, while the estimated number of first-born babies was 1208, calculated from the number of first-born babies in 2006 in Vantaa. To obtain an absolute risk reduction of 10%, which was considered clinically significant, about 726 children needed to be recruited into the questionnaire study. We exceeded this number of patients during the inclusion period; parents of 804 children accepted the offer to participate and signed the informed consent form of the study on behalf of their child. The estimated amount of dropouts was 18%, and the observed amount was 20%.

### 2.4. Training

The dental professionals involved in the counseling of young children were given training for the study and also to improve the quality of oral health promotion in the PDS of Vantaa. The training to perform MS plaque testing was organized in small groups in eleven daycare centers for young children in 2007. The training on how to interpret the results was arranged and repeated in several separate sessions, because the dental professionals first informed the parents of their own estimate of the test result. Thereafter, the parents were informed if the MS score was altered after the judgment of the author (IA) who interpreted all tests for the present study blinded. In order to maintain the reliability of MS testing, the author responsible for interpreting all tests was trained by 100 MS plaque tests with a senior researcher (KP) in the beginning of 2008. The tests were randomly selected from the 1000 test that had been performed. In case of differences of opinion, these were discussed, with the emphasis on avoiding the overregistration of colonies. 

The dental professionals were trained to use the new oral health programs for the study groups F and X through lectures and written instructions [17]. The transtheoretical model (TTM) [11] was selected as the theoretical framework, and the stages of change were combined with the method of motivational interviewing (MI) [12, 13] in preventive counseling of all programs. 

The training on how to observe dental decay and control the progression of caries lesions was arranged in a one-day session prior to the children's two-year visits in 2009. The session was followed by individual exercises in groups of three participants in the dental clinics of primary schools. The dental professionals were given individual feedback on their observations of dental decay and the use of counseling methods at the end of each exercise. The information from all sessions was repeated later in a one-afternoon session. 

### 2.5. Interventions

The oral health interventions were carried out at children's routine oral health visits from 6 to 36 months of age for all the children visiting the PDS. The first visit was at the age of 6–12 months. About ten percent of the children were assessed to have a high caries risk and they had an extra visit at the age of 18 months. The risk factors that were agreed upon by a team of dental professionals included visible plaque or caries on child's teeth, sugary drink as a thirst-quencher, bedtime feeding, special health care need [18, 19], parent's nonchalant attitude to dental care, serious social problems in the family, or an immigrant family background [20]. The following visit for all children was at the age of 24–36 months. 

The children were treated in the same way in all groups. The main differences between the groups were that in group F the parents were given counseling on their own oral hygiene, and in group X the parents were given counseling on their diet, whereas in the routine program, there were no interventions for the parents. The new family-based oral health promotion programs for the study groups F and X were created by a team of dental hygienists and dentists. 

The routine program in use earlier in the Vantaa PDS included comprehensive advice on children's oral health. The counseling included regular oral hygiene, that is, tooth brushing with fluoride-containing toothpaste twice a day, starting from the appearance of the first tooth. It also included advice on a healthy diet with proper timing and composition of meals, avoiding sugary snacks, as well as a recommendation to use xylitol products on a regular basis, 5 grams per day. In order to delay the transmission of MS from mother to child, the parents were advised to avoid tasting from the child's spoon, to avoid cleaning the child's pacifier in their own mouth, and to use xylitol products regularly. Each child was given a toothbrush on the first visit and a brochure containing information about oral health care at home. 

In group F (oral hygiene and fluoride), on the child's first visit to the PDS, the parents were given counseling on how to brush their own teeth. Tools for cleaning the interdental spaces of the parent were shown, and he or she was given a toothbrush, one tube of fluoride toothpaste, and samples of dental floss, sticks, or interdental brushes. Goals for cleaning the teeth of both parent and child were set together and written down in a form that was given to the parent. On the child's second visit at the age of two years, the dental hygienist brought up these goals again and encouraged the parent to follow them. 

In group X (diet and xylitol), the special elements added for the parents were a properly timed and healthy diet and the regular use of xylitol. On the child's first visit, the parent was asked to fill in a one-day diary concerning his or her own diet. The dental professional pointed out the frequency of meals and snacks and discussed the related pH drop, as well as suggesting the use of 5 grams of xylitol per day. A packet of xylitol mints (Xylisuu, Fennobon Oy, PL 4, 00941 Helsinki) was given to the child and some samples of chewing gum, sweetened only by xylitol, to the parents. Pictures demonstrating the sugary contents of some snacks and soft drinks were shown from an illustrated catalog. Goals for maintaining oral health were set together and entered in a form. On the child's dental visit at the age of two years, the goals were reviewed and the parents were encouraged to follow them.

During the present study, each of the 28–35 dental professionals carried out the counseling individually. One of the authors (IA) read the recordings in the electronic patient database to find out how thoroughly the interventions had been implemented and recorded in groups F and X. Individual variation was found; in some cases the interventions and/or registrations may have been incomplete. 

### 2.6. Clinical Examinations and MS Determination

The children, accompanied by their parents, were clinically examined in various health clinics of the Vantaa PDS. The dental hygienists or the in-service trained dental nurses working in the clinic performed the examinations. The information from them including the number of decayed teeth or teeth with distinct visual changes in enamel corresponding to ICDAS [21] values 2 to 6, was recorded on the database. 

Plaque testing of the two-year-olds was started in Vantaa in January 2008. The tests were carried out at the children's regular two-year visits. The recommended age for testing was 2–2.5 years, and the inclusion limits were 24–36 months. The presence of MS was determined by the Strip Mutans Dentocult SM test (Orion Diagnostica Oy, P.O. Box 83, 02101 Espoo, Finland). The MS tests were taken from plaque because when assessing caries risk in young children, apparently the sensitivity and specificity are better in plaque testing than in saliva testing [9]. Samples of plaque were obtained using four separate microbrushes and applied on the strip from four predestined tooth surfaces: the interdental spaces and the gingival margin of upper incisors, upper molars, lower incisors, and lower molars. The strips were incubated at 35–37°C for a minimum of 48 hours. The incubators were calibrated every six months. The dental professionals dried and interpreted the strips according to the manufacturer's instructions and informed the parents by letter or by phone of the test result. The dried strips were then sent to one of the authors (IA) who interpreted all MS tests blinded.

### 2.7. Data Collection and Management

The data were retrieved from the patient records and the database of the questionnaires. The scores 0–3 of MS tests were dichotomized into MS 0 (negative) and MS 1+ (positive). Incidence of caries, including the number of decayed teeth and teeth with distinct visual changes in enamel, was dichotomized as 0 for no caries and 1 for the other values. To enable a blind set-up in the comparison of birth cohorts, the MS tests taken from January 2008 to June 2009 and a random sample of 800 tests taken in 2009-2010 were reinterpreted, mixed up in random order with all tests carried out in 2011, and used for the present analyses. The dental professionals collected the questionnaires and sent them to the author (IA) who entered the information into the database. 

For the families of the questionnaire study, mother's and father's ages were dichotomized into younger (born 1980 or later) and older (born before 1980), and parental levels of education as basic (basic, vocational, or high school) and advanced (college or university degree). Parents' habits of cleaning the child's pacifier in their own mouth and tasting food from the child's spoon were dichotomized into 0 (seldom or never) and 1 for the other values. The child's reported health habits of tooth brushing and fluoride use were dichotomized into 1 (at least 2 times/day) and 0 for the other values. Having snacks between meals was dichotomized into 0 (seldom or never) and 1 for the other values, and xylitol use into 1 (at least 3 times a day) and 0 for the other values. The child's use of probiotics was dichotomized into 0 (not at all) and 1 (used for one month or more).

### 2.8. Data Analysis

The main outcome measure of the study was the colonization of MS bacteria in the dental plaque of the children aged 24–36 months. The MS values were cross-tabulated and compared by cohort and intervention group. Within the questionnaire study, the parental information and the child's reported health habits were cross-tabulated by intervention group. The association of parental information and child's reported health habits with MS were analyzed using univariate logistic regression analyses. Multivariate logistic regression analyses with manual backward elimination were also used. All independent variables for which the regression coefficient did not reach statistical significance were eliminated one by one. Intervention group and area (three matched areas in Vantaa) were included in regression models as confounders. The statistical software used was PASW statistics 20.0, and the level of statistical significance was set at *P* < 0.05.

## 3. Results

On average, 60% of the families visited the PDS when the child was six months old. At the two-year examination, the percentages were 66 and 76% in the 2006 and 2008 birth cohorts, respectively. The MS tests were taken from 57% in the 2006 birth cohort, from 77% in the 2008 birth cohort (including the first-born children), and from 95% in the questionnaire study of first-born children.

In the 2008 and 2006 birth cohorts, colonization of MS was found only in few children (Table 1). A positive trend was found between the 2006 and 2008 birth cohorts in relation to the MS score; the percentages of negative MS scores were 85 and 89%, respectively, (*P* = 0.011). The lowest scores for MS in the 2008 birth cohort were found in the control group (*P* = 0.011) (Table 1). Altogether, 4% in the 2008 birth cohort and 5% in the 2006 birth cohort had experienced caries, no missed or filled teeth were found.

In the questionnaire study of first-born children, 52% were boys, 48% girls, and thirty children (5%) were not tested for MS. No group differences were found in the MS colonization. In oral health habits, two group differences were found: the parents tasting food from the child's spoon and the child's frequency of using xylitol products. In the F group, 61% of parents reported not having tasted food from the child's spoon compared to 48% and 55% of groups X and C, respectively, (*P* = 0.041), while 56% of children in the X group were reported to have used xylitol at least three times a day compared to 48% and 42% in the F and C groups, respectively, (*P* = 0.029). The reported frequency of tooth brushing twice a day was about 60%, and about half of the children were reported to have had snacks between meals seldom or never (Table 2). The parents of 38 children did not return for the two-year questionnaire, and six of these children were not tested for MS either.

The advanced education level of mother (*P* = 0.006) and father (*P* = 0.026), child's tooth brushing (*P* = 0.031), as well as child's use of xylitol at least three times a day (*P* = 0.006) associated with negative scores in the MS test. In multivariate analysis, father's advanced education level (*P* = 0.044) and child's xylitol use at least three times a day (*P* = 0.014) remained in the final model. No associations were found between the presence of caries and the parent- or child-related factors (Table 3). 

No serious adverse effects of using xylitol products were detected during the study. The parents reported some adverse effects; in 11 cases, parents reported that the child did not want to or was not able to chew xylitol mints or chewing gum. In 21 cases, parents reported gastrointestinal complaints; these included flatulence, diarrhea, and, in one case, constipation. In two cases, these symptoms were reported to have disturbed the child only in the beginning of xylitol use. 

## 4. Discussion

The observed reduction in the proportion of MS-colonized children between the 2006 and 2008 cohorts suggests that the training and supporting of professionals may have clinical benefits. The finding that there were no group-related differences within the 2008 cohort indicates that the addition of parental self-care to the comprehensive routine prevention in early childhood does not further improve the results. The findings of the multifactorial analyses suggest that father's education level and child's xylitol use associate with child's negative MS scores.

The prevalence of MS colonization, as well as of dental caries, in two-year-olds was lower than anticipated on the basis of earlier findings in Finland [16]. The favorable result in the control group most probably indicates that our matching was not as successful as we hoped; within a more homogenous first-born children sample, this difference did not exist. The low incidence of caries is most probably the main reason for our not finding any outstanding differences between the programs in relation to caries. An additional reason may be the fact that the training was given to all the dental professionals, not only to those involved in the new programs. This kind of training improves the skills and knowledge of personnel, and most probably increases the quality of care given, but inevitably reduces program-related differences. The present finding is, however, in line with studies in Belgium [22], in Finland [10], and in Sweden [23] in which oral health intervention programs did not result in a significant reduction in caries prevalence. 

In the 2008 birth cohort, the number of families that visited the PDS when the child was two years old was higher compared to the historic control group, the 2006 birth cohort. Thus, the 2008 birth cohort probably included a larger share of families with children at high risk of caries, a fact that should have changed the main outcome measures in the 2008 birth cohort for the worse. However, the 2008 birth cohort turned out better than that of 2006; at two years of age, there were more negative MS scores in the birth cohort of 2008 than in 2006. A sensitivity analysis was performed to assess whether the results may have been biased because of missing data. In the best case scenario, if all missing MS scores had been negative, there would be no difference between the cohorts. In the worst case scenario, if all missing MS scores had been positive, the difference (19%) between the cohorts would be statistically significant. In a third scenario, we hypothesized that the percentage of negative results in the missing cases of both cohorts would resemble those in the fathers' basic level of education (84%). In that scenario, the difference (2%) between the cohorts was statistically significant, although clinically minimal (*P* = 0.018).

The finding that father's advanced education level associates with the child's negative MS scores is in line with Ersin et al.'s [24] study of early childhood caries. In their study, the mother's lower level of education was a strong risk indicator for the colonization of caries-related micro-organisms. In the study of Meurman et al. [10], early colonization of MS was found to be associated with the socioeconomic status of the family. A systematic review of parental influence on the development of dental caries in children aged 0–6 years old [25] suggests that lower socioeconomic status are associated with higher prevalence or severity of caries in young children, and that the low level of parental education is associated with a higher risk for caries. In the present study, most of the univariate associations were found with the education level of both parents but, in multivariate analysis, father's education level proved to be more important than mother's education level.

In the present study, there were two group-related differences found in oral health habits; the lower proportion of the habit of tasting food from the child's spoon by parents in group F, and the child's reported higher use of xylitol products in group X. The findings are in accordance with the programs used; in program F, the procedure of pointing out the biofilm on the parents' teeth might have increased their awareness of having contagious bacteria in their mouth. In program X, the discussion on the frequency of meals and snacks, and the related pH drop might have motivated the parents to use xylitol also for their children. The benefits of xylitol in oral health promotion have been shown in numerous studies starting from the 1970s. According to The European Food Safety Authority [26], a total daily dose of 2-3 grams of chewing gum sweetened with 100% xylitol at least three times per day after meals is required for clinical effect. The first-born children in the questionnaire study use xylitol products very often, especially those of fathers with an advanced level of education. The use of xylitol products is common in Finland, and the use has been promoted for years; at the present, it is a generally accepted smart habit [27]. According to the present study, the use of xylitol three times a day is quite well tolerated by children. 

The MS testing continued for almost four years, which constituted a risk for the reliability of the main outcome measure. For this reason, all the first-year tests and a large proportion of other tests were reinterpreted for the present analyses, randomly mixed up with all the last-year tests. This enabled a blind set-up in the comparison of birth cohorts, and increased the reliability of the results.

In the 2008 birth cohort, 77% of the children that visited the PDS were tested for MS in comparison with 95% of first-born children in the questionnaire study. The high proportion may be the result of a study effect, concerning the parents of first-born children, as well as the dental professionals involved. 

In this study, we wanted to create something new for the interventions; in addition to counseling on children's oral health, we also wanted to advise the parents themselves, with the intention of promoting the oral health of the whole family. This addition of the parental aspect was not found more effective than the routine program. According to the opinions of the dental professionals, the parents accepted the interventions well. Before the implementation of this study, the dental professionals were given a great deal of instruction in several aspects of oral health promotion, including introducing the use of the transtheoretical model and motivational interviewing, and training in their use was arranged [17]. Within the organization, the efforts added to the counseling of young children may have increased respect for early childhood oral health promotion and the professionals responsible for it; this might also have had an effect on their attitudes towards the work. This is in line with the study of primary nurses' performance, which emphasizes the role of supportive management [28]: a higher level of supervisory support for the nurses resulted in higher performance at the workplace compared to the performance of the nurses receiving a lower level of support. The managers were found to be important [29] in supporting the nurses to create a culture of shared responsibility of evidence-based work, and to achieve a real change process in patient's education.

In conclusion, the present findings suggest that training and supporting the professionals in health education is more important than adding parental self-care to programs for young children. Instead of a program effect, the father's advanced education level and the child's use of xylitol were found to be associated with negative MS scores in the child. The counseling of young families might be best carried out by the routine program; focusing only on the few main issues of the child's oral health promotion.

## Figures and Tables

**Table 1:** First-born children, born in 2008, in comparison with the 2008 and 2006 birth cohorts; percentages of negative MS scores in 24–36-month old by group.

	First-born children born in 2008		The 2008 birth cohort		The 2006 birth cohort*	
	Total *n*	%	Total *n*	%	Total *n*	%
MS score negative (=0)	617	91	1525	89**	955	85**
F group (oral hygiene and fluoride)	242	91	686	87	419	83
X group (diet and xylitol)	176	89	390	88	231	89
Control group	199	92	449	93	305	86
*P*		0.658		**0.011**		0.129

*The same areas where the 2008 birth cohort was given interventions.

**P = 0.011.

**Table 2:** Parental background information and child's reported health habits in first-born children at the age of 24–36 months; percentages within groups.

	Total *n*	Oral hygiene and fluoride F	Diet and xylitol X	Control C	*P*
*Parents *					
Mother born before 1980	644	56	66	60	0.088
Father born before 1980	625	71	76	72	0.462
Mother's advanced education level	601	62	70	69	0.148
Father's advanced education level	575	51	54	58	0.269
Parents have not cleaned child's pacifier in their own mouth	597	94	95	95	0.738
Parents have not tasted food from child's spoon	611	61	48	55	**0.041**
Mother's use of xylitol at least 3 times/day	605	19	20	16	0.563
*Child *					
Tooth brushing at least 2 times/day	613	65	59	58	0.293
Fluoride use at least 2 times/day	613	65	60	59	0.394
Snacks between meals seldom or never	609	49	49	56	0.234
Xylitol at least 3 times/day	613	48	56	42	**0.029**
Use of probiotics products	558	58	46	54	0.062

**Table 3:** Results of the logistic regression models of parent- and child-related factors on the presence of MS among first-born children at the age of 24–36 months with intervention group and area as confounders.

	MS score negative				
	Crude OR	*P*	OR	95% CI	*P*
Mother born before 1980	0.67	0.153			
Father born before 1980	0.77	0.407			
Mother's advanced education level	0.44	**0.006**			
Father's advanced education level	0.48	**0.026**	0.51	0.27–0.98	**0.044**
Parents have not cleaned pacifier in their own mouth	0.49	0.175			
Parents have not tasted food from child's spoon	0.86	0.614			
Mother's use of xylitol at least 3 times/day	0.89	0.764			
Child's tooth brushing at least 2 times/day	0.53	**0.031**			
Child's fluoride use at least 2 times/day	0.61	0.096			
Child's snacks between meals seldom or never	0.91	0.750			
Child's use of xylitol at least 3 times/day	0.41	**0.006**	0.41	0.20–0.84	**0.014**
Child's use of probiotic products	0.72	0.290			
Gender (male = 1)	0.74	0.281			

## References

[1] Pienihäkkinen K, Jokela J, Alanen P (2005). Risk-based early prevention in comparison with routine prevention of dental caries: a 7-year follow-up of a controlled clinical trial; clinical and economic aspects. *BMC Oral Health*.

[2] Plutzer K, Spencer AJ (2008). Efficacy of an oral health promotion intervention in the prevention of early childhood caries. *Community Dentistry and Oral Epidemiology*.

[3] Joensuu T (2009). *Cumulative Costs of Caries Prevention and Treatment in Children—With Special Reference to Work Division and Cohort Effect*.

[4] SIGN Scottish Intercollegiate Guidelines Network. http://www.sign.ac.uk/guidelines/fulltext/83/index.html.

[5] Kaypa hoito—Karies (hallinta). http://www.kaypahoito.fi/web/kh/suositukset/naytaartikkeli/tunnus/hoi50078.

[6] Kressin NR, Nunn ME, Singh H (2009). Pediatric clinicians can help reduce rates of early childhood caries: effects of a practice based intervention. *Medical Care*.

[7] Gizani S, Papaioannou W, Haffajee AD, Kavvadia K, Quirynen M, Papagiannoulis L (2009). Distribution of selected cariogenic bacteria in five different intra-oral habitats in young children. *International Journal of Paediatric Dentistry*.

[8] Takahashi N, Nyvad B (2008). Caries ecology revisited: microbial dynamics and the caries process. *Caries Research*.

[9] Thenisch NL, Bachmann LM, Imfeld T, Leisebach Minder T, Steurer J (2006). Are mutans streptococci detected in preschool children a reliable predictive factor for dental caries risk? A systematic review. *Caries Research*.

[10] Meurman P, Pienihäkkinen K, Eriksson AL, Alanen P (2009). Oral health programme for preschool children: a prospective, controlled study. *International Journal of Paediatric Dentistry*.

[11] Prochaska JO, Redding CA, Evers KE, Glanz K, Rimer BK, Lewis FM (2002). The transtheoretical model and stages of change. *Health Behavior and Health Education: Theory, Research, and Practice*.

[12] Resnicow K, McMaster F (2012). Motivational Interviewing: moving from why to how with autonomy support. *International Journal of Behavioral Nutrition and Physical Activity*.

[13] Harrison RL, Veronneau J, Leroux B (2012). Effectiveness of maternal counseling in reducing caries in Cree children. *Journal of Dental Research*.

[14] Miller WR, Rose GS (2009). Toward a theory of motivational interviewing. *The American Psychologist*.

[15] Yevlahova D, Satur J (2009). Models for individual oral health promotion and their effectiveness: a systematic review. *Australian Dental Journal*.

[16] Pienihäkkinen K, Jokela J, Alanen P (2004). Assessment of caries risk in preschool children. *Caries Research*.

[17] Arpalahti I, Jarvinen M, Suni J, Pienihäkkinen K (2012). Acceptance of oral health promotion programmes by dental hygienists and dental nurses in public dental service. *International Journal of Dental Hygiene*.

[18] Warren JJ, Weber-Gasparoni K, Marshall TA (2008). Factors associated with dental caries experience in 1-year-old children. *Journal of Public Health Dentistry*.

[19] Tinanoff N, Reisine S (2009). Update on early childhood caries since the surgeon general’s report. *Academic Pediatrics*.

[20] Wigen TI, Espelid I, Skaare AB, Wang NJ (2011). Family characteristics and caries experience in preschool children. A longitudinal study from pregnancy to 5 years of age. *Community Dentistry and Oral Epidemiology*.

[21] ICDAS foundation International caries detection and assessment system. http://www.icdas.org/.

[22] Vanobbergen J, Declerck D, Mwalili S, Martens L (2004). The effectiveness of a 6-year oral health education programme for primary schoolchildren. *Community Dentistry and Oral Epidemiology*.

[23] Sundell AL, Ullbro C, Koch G (2013). Evaluation of preventive programs in high caries active preschool children. *Swedish Dental Journal*.

[24] Ersin NK, Eronat N, Cogulu D, Uzel A, Aksit S (2006). Association of maternal-child characteristics as a factor in early childhood caries and salivary bacterial counts. *Journal of Dentistry for Children*.

[25] Hooley M, Skouteris H, Satur J, Kilpatrick N (2012). Parental influence and the development of dental caries in children aged 0–6 years: a systemic review of the literature. *Journal of Dentistry*.

[26] EFSA Opinion of the Scientific Committee/Scientific Panel: Xylitol chewing gum/pastilles and reduction of the risk of tooth decay—Scientific substantiation of a health claim related to xylitol chewing gum/pastilles and reduction the risk of tooth decay pursuant to Article 14 of Regulation (EC) No 1924/2006[1]—Scientific Opinion of the Panel on Dietetic Products, Nutrition and Allergies.

[27] Tolvanen M, Lahti S, Poutanen R, Seppä L, Pohjola V, Hausen H (2009). Changes in children’s oral health-related behavior, knowledge and attitudes during a 3.4-yr randomized clinical trial and oral health-promotion program. *European Journal of Oral Sciences*.

[28] Drach-Zahavy A (2004). Primary nurses’ performance:role of supportive management. *Journal of Advanced Nursing*.

[29] Bergh AL, Persson E, Karlsson J, Friberg F (2013). Registered nurses' perceptions of conditions for patient education—focusing on aspects of competence. *Scandinavian Journal of Caring Science*.

